# Health-related quality of life among families of children with severe bronchopulmonary dysplasia

**DOI:** 10.1038/s41372-025-02468-x

**Published:** 2025-11-04

**Authors:** Kathryn Farrell, Sara B. DeMauro, Kathleen Gibbs, Heidi Morris, Kathleen Nilan, Katharine P. Callahan

**Affiliations:** 1 The Perelman School of Medicine at the University of Pennsylvania, Philadelphia, PA USA; 2 Division of Neonatology, Children’s Hospital of Philadelphia, Philadelphia, PA USA; 3 Department of Medical Ethics and Health Policy, The Perelman School of Medicine at the University of Pennsylvania, Philadelphia, PA USA

**Keywords:** Outcomes research, Quality of life

## Abstract

**Objectives:**

We aimed to profile the family impact, specifically parental health-related quality of life (HRQoL) and family functioning, of children discharged from a quaternary referral program with the most severe forms of bronchopulmonary dysplasia (BPD).

**Study design:**

We collected cross-sectional data through telephone interviews with 282 families of children aged 18 months to 11 years who had been discharged from a BPD referral program.

**Results:**

Parental HRQoL and family functioning were not associated with the child’s age, despite improvements in the children’s health and respiratory morbidity with age. Many medical issues negatively impacted parental HRQOL and family functioning. The health outcome most strongly associated with decreased parental HRQoL and family functioning was an autism diagnosis, followed by use of feeding tube.

**Conclusions:**

Among families of children discharged from a quaternary BPD program, family impact was not associated with child age and was impacted by a wide range of health outcomes in their children.

## Introduction

Bronchopulmonary dysplasia (BPD) has a substantial impact on children’s families during the initial hospitalization and following discharge. One study conducted during children’s first two years of life showed that all domains of health-related quality of life (HRQoL) included in the PedsQL Family Impact Module were significantly, negatively affected by taking care of a child with BPD [1]. This impact is not surprising in light of the long-term sequelae and high rates of technology dependence in this population. Infants with BPD experience significantly increased healthcare needs, including supplemental oxygen, nutrition, therapies, specialist visits, and medications, compared to preterm infants without BPD and term controls [2, 3]. Approximately half of infants with BPD will be re-admitted to the hospital during childhood due to respiratory problems and most require more frequent outpatient visits [4, 5]. Infants with BPD experienced greater impacts on cognitive and motor development and are more likely to be enrolled in special education compared to controlled VLBW infants without BPD [6]. The effects of BPD on parental HRQoL and family functioning are found to be long-lasting. One study found that respiratory morbidity in premature children continues to affect the parent’s quality of life and family functioning beyond infancy in school aged children [7]. Yet, in a number of studies the negative impact of BPD on families is shown to decrease over time [1, 7–9].

Increasingly, patients with the most severe forms of BPD survive [10]. Our group recently reported on the outcomes of patients discharged from a referral BPD program who have among the most severe sequalae reported. Forty two percent of patients had a tracheostomy at some point. Ninety eight percent had individualized education plans (IEPs) in school, and thirteen percent had a diagnosis of autism [11]. The NICHD Neonatal Research Network found that rates of re-hospitalization, pulmonary medication use and incidence of poor outcomes requiring medical intervention increased with the severity of BPD [12]. In other diseases, severity of the illness correlates with its ramifications for families [13, 14]. For example, one study found that mild perinatal strokes had no impact on family quality of life, while strokes with severe medical sequelae impacted parental depression, marital satisfaction, quality of life and family functioning [15]. Severe BPD is expected to more negatively impact families’ quality of life and functioning due to the associated demands of caring for a child and need for medical technology. Yet, little is known about the specific nature of this impact or how impact varies between children of different ages.

As part of a larger cross-sectional study on the outcomes of children with severe BPD, we assessed the relationship between the specific health resource needs of the child and parental HRQoL, differences in parental HRQoL based on child’s age, and the subcategories within parental HRQoL that are particularly impacted. Additionally, we investigated how the parents’ HRQoL and family functioning differed for children with and without tracheostomies. A more complete understanding of how severe forms of BPD impact parental HRQoL and family functioning across childhood will help clinicians target support for families.

## Methods

### Participants and recruitment

We contacted parents or guardians of children who were cared for between May 2010 and November 2020 in the Neonatal Chronic Lung Disease (NeoCLD) Program at Children’s Hospital of Philadelphia (CHOP). This program treats patients who were referred from across the United States with the most severe forms of BPD. Seventy percent of transfers are from outside of the network of hospitals directly affiliated with CHOP. The CLD Program maintains a clinical registry of all program participants, which we used to identify eligible participants. Additional inclusion criteria for the current study were: the child was still alive and was 0–12 years old when the questionnaires were administered. There was no inclusion criteria based on BPD grade. We did not contact the families of deceased patients, but the age and cause of death were recorded from the electronic medical record. We used medical translators for non-English speaking participants to assist in completing the questionnaires. We contacted all eligible participants through phone calls. After three attempted phone calls without contact, we mailed a letter to the address on file. The CHOP Institutional Review Board deemed this study exempt (Category 2).

### Data collection

A multidisciplinary group including physicians, nurses, social workers, and physical therapists, all with a focus on the clinical care and research related to children with severe BPD, selected validated instruments and additional questions to administer to parents. The group selected instruments that are valid across a wide age range, in children with a wide range of abilities, and appropriate for telephone administration. The final list of instruments collected information about:

1. Quality of life as reflected in the PedsQL Inventory
2. PedsQL Family Impact Module (PedsQL FIM) [16]
3. Respiratory symptoms as recorded by the International Study of Asthma and Allergies in Childhood (ISAAC) [17–19]
4. Child’s development as reflected in the Adaptive Behavior Assessment System (ABAS), 3rd Edition [20]

The group also designed tailored questionnaires to collect family demographic characteristics and data on the child’s health since discharge. They included the question: “In general, how would you describe your child’s health?” which parents were asked to respond to on a 5-point Likert scale poor to excellent.

We gathered most data from parent report during the interview. The CHOP electronic medical record and the NeoCLD Program registry were used to obtain additional demographic information, baseline perinatal characteristics of the child, and information about inpatient hospitalizations and outpatient care. When discrepant, parent reports were trusted over medical records. For example, if a parent reported a diagnosis of autism from an outside provider, the child was marked as having autism even if not indicated in our medical record. Per the 2019 NRN definition, BPD grade was based on level of respiratory support at 36 weeks [19]. The PedsQL forms include items scored on a 0–100 scale, with higher scores designating better quality of life. The PedsQL FIM includes 36 questions in 8 domains focused on concerns or difficulties the parents or family may have had due to their child’s health within the last month. The total score is calculated by averaging the eight domains: physical, emotional, social, and cognitive functioning, communication, worry, daily activities, and family relationships. The parent HRQoL summary score is calculated by averaging the physical, emotional, social, and cognitive functioning scores. The family functioning summary score is calculated by averaging the daily activities and family relationships scores.

### Statistical analysis

We collected all data in a REDCap database. Only the study team had access to the data and subjects were assigned a unique participant number to protect confidentiality. We exported de-identified data to STATA v16.1 (College Station, TX) for analysis. For all variables, we calculated the percentage of children affected, mean values, and standard deviations. We assessed normality before selecting statistical tests. We examined the correlations between PedsQL FIM and several pre-specified primary outcomes using Wilcoxon rank sum or Spearman’s rank correlation coefficient, as appropriate, to assess for significant relationships and then used regression analysis to assess the magnitude of the correlations. To show results in association with ages of the children, the cohort was split into two groups: aged younger than five years or aged five years and older. Selecting the age of five allowed the young and older cohort to be similar in size, as well as accounted for a significant change in the lives of many families as this is the typical age in which children begin school. We prospectively designated the following variables as primary outcomes: respiratory support (yes or no), tracheostomy (ever placed), current medicines (number), current respiratory medicines (number), wheezing (ever in past 12 months), rehospitalizations (lifetime number), hearing or vision impairment (yes or no), poor weight gain, walking alone, cerebral palsy (CP, yes or no), developmental delay, autism, outpatient therapies (number of services), and parent-reported child health. For parent-reported health, we re-scaled the Likert rating of overall health to a 100-point scale to parallel the HRQoL metric and assessed the correlations between parental-reported health and PedsQL summary scores. We used linear regression to evaluate correlations between PedsQL FIM and child’s current age. We compared patient characteristics between respondents and non-respondents using *t*-tests. We did not correct for multiple comparisons as these analyses are exploratory in nature.

## Results

Between June 2021 and July 2022, our team collected information via phone interview on 568 children who were cared for by the NeoCLD Program at CHOP and survived to discharge. Eighty-nine of these children (16%) died after NICU discharge and were excluded from the study. We contacted 479 families, of whom 282 participated in the study (59% overall, 67% of those with whom we established contact). Birthweight, gestational age, gestational age at discharge, and child age at the time of attempted contact did not differ between respondents and nonrespondents (Supplementary Table 1). At the time of follow-up, the mean age in our cohort was 5.7 years (Table 1, SD 2.9). Our group previously published data that demonstrated children’s respiratory and developmental morbidities for this cohort were worse than any previously reported in the literature in terms of respiratory and developmental status [11].

**Table 1:** Characteristics of the study sample and family impact module scores [11].

Characteristic	Percentage or Mean (SD) *n* = 282 (unless noted)
*Child demographic characteristics*	
Female sex	36.9%
*Race*	
Asian	2.1%
Black or African American	33.1%
Indian	0.4%
Multi-Racial	6.4%
Native Hawaiian or other Pacific Islander	0.4%
White	35.9%
Other	19.2%
Refused	2.1%
*Ethnicity*	
Hispanic or Latino	12.1%
Not Hispanic or Latino	85.4%
*Birth/NICU characteristics*	
Gestational age (weeks)	26.0 (2.6)
Birthweight (grams)	839.2 (458.8)
*Respiratory support at 36 weeks PMA*	
No respiratory support (no BPD)	1.7%
≤2 L nasal cannula (Grade 1 BPD)	5.8%
Noninvasive support >2 L (Grade 2 BPD)	38.4%
Intubated (Grade 3 BPD)	54.1%
PMA at NICU discharge (weeks)	61.6 (14.3)
*Respiratory support at discharge*	
No respiratory support	17.9%
≤2 L nasal cannula	31.1%
Noninvasive support >2 L nasal cannula	9.7%
Tracheostomy	41.4%
*Follow-up*	
Child age at follow-up (years)	5.7 (2.9)

*PMA* Post-menstrual age.

The mean total score for PedsQL Family Impact Module (FIM) in the entire cohort was 76.7 (SD 22.3). Worry and daily activities were the lowest-scoring subsections. The highest-scoring subsections were cognitive functioning and family relationships (Table 2). The lowest scoring (highest concern) questions amongst all subsections were: I worry about my child’s future, I feel that others do not understand my family’s situation, it’s hard to find time for social activities, and I feel tired in the morning and throughout the day (Table 3). The questions with the highest scores (least concern) amongst all subsections were: I feel sick to my stomach, I feel physically weak, it is hard for me to tell doctors and nurses how I feel, I feel helpless or hopeless, and I feel angry.

**Table 2:** Average PedsQL family impact module scores.

Parent and family quality of life (PedsQL v 2.0 family impact module)	
Family impact module total score	76.7 (22.3)
Subscore: physical	76.5 (23.6)
Subscore: emotional	78.1 (22.9)
Subscore: social	72.1 (28.7)
Subscore: cognitive	82.3 (22.6)
Parental HRQoL summary score	77.5 (21.3)
Subscore: daily activities	68.4 (30.6)
Subscore: family relationships	81.6 (22.7)
Family function summary score	75.8 (19.6)
Subscore: worry	68.3 (23.2)
Subscore: communication	74.3 (24.4)

**Table 3:** Ten lowest scoring questions on the PedsQL family impact module.

Question	Mean (SD) *N* = 281
I worry about my child’s future	44.4 (37.4)
I feel that others do not understand my family’s situation	56.9 (36.2)
It’s hard to find time for social activities	61.8 (38.8)
I feel tired when I wake up	63.4 (36.1)
I feel tired during the day	63.8 (36.1)
I have difficulty finding time to finish household tasks	66.7 (35.7)
I feel anxious	67.8 (34.6)
I worry about the side effects of my child’s medications/medical treatments	68.1 (34.4)
Family activities take more time and effort	68.9 (35.8)
I feel too tired to finish household tasks	69.6 (34.1)

The average summary scores and the respective subsections of the PedsQL did not change with the age of the child. Additionally, most of the ten lowest scoring questions (highest concern) remained consistent between parents of toddlers aged younger than five years old, and parents of children aged five years of age or older (Table 4). However, parents with children under the age of five years more often reported having difficulty finding time to finish household tasks and feeling too tired to finish household tasks. Parents of children older than five years more often reported worrying about how others will react to their child’s condition and feeling frustrated.

**Table 4:** Ten lowest scoring questions across age groups.

All ages		Age under 5 years (*n* = 140)		Age 5 years or older (*n* = 142)	
Question	Mean (SD) *N* = 280	Question	Mean (SD) *N* = 140	Question	Mean (SD) *N* = 142
I worry about my child’s future	44.4 (37.4)	I worry about my child’s future	49.1 (37.1)	I worry about my child’s future	39.8 (37.3)
I feel that others do not understand my family’s situation	56.9 (36.2)	I feel tired during the day	58.8 (35.3)	I feel that others do not understand my family’s situation	54.6 (39.6)
It’s hard to find time for social activities	61.8 (38.8)	I feel that others do not understand my family’s situation	59.8 (32.5)	It’s hard to find time for social activities	63.6 (40.3)
I feel tired when I wake up	63.4 (36.1)	I feel tired when I wake up	60.2 (35.3)	I feel tired when I wake up	66.4 (37.0)
I feel tired during the day	63.8 (36.1)	It’s hard to find time for social activities	60.4 (37.1)	I feel anxious	66.4 (36.5)
I have difficulty finding time to finish household tasks	66.7 (35.7)	Difficulty finding time to finish household tasks	63.8 (35.1)	I worry about the side effects of my child’s medications/medical treatments	68.1 (34.9)
I feel anxious	67.8 (34.6)	I feel too tired to finish household tasks	68.0 (33.5)	Family activities take more time and effort	68.1 (37.3)
I worry about the side effects of my child’s medications/medical treatments	68.1 (34.4)	I worry about the side effects of my child’s medications/medical treatments	68.4 (33.9)	I worry about how others will react to my child’s condition	68.3 (35.1)
Family activities take more time and effort	68.9 (35.8)	I feel anxious	69.3 (32.6)	I feel tired during the day	68.5 (36.5)
I feel too tired to finish household tasks	69.6 (34.1)	Family activities take more time and effort	69.8 (34.1)	I feel frustrated	69.4 (32.

**Fig. 1:**
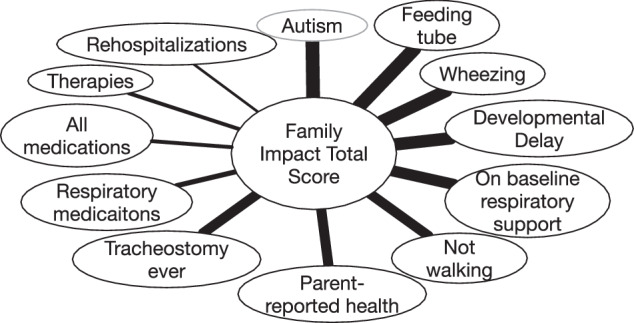
Correlations between child outcomes and total family impact module score. Line thickness represents strength of correlation.

In regression analysis, the following child medical and developmental characteristics and outcomes were associated with worse PedsQL FIM score (ordered from greatest to least impact): Autism, tube feeds in the past year, wheezing in the past year, developmental delay, need for baseline respiratory support, not walking, parent-reported health, having had a tracheostomy, number of respiratory medications, number of total medications, number of outpatient therapies, and number of rehospitalizations (Fig. 1). Autism, tube feeds, and history of recent wheezing were associated with 11.3, 11.1, and 10.4 point reductions in PedsQL FIM score, respectively (Remainder of correlation coefficients provided in Supplementary Table 2). A diagnosis of CP, poor weight gain, vision and hearing impairment were not associated with PedsQL FIM.

## Discussion

We present parental HRQoL and family function outcomes for a referral cohort of children with BPD, the most severe cohort reported on a number of respiratory and developmental outcomes [11]. Compared to previous studies of families impacted by BPD that used the PedsQL FIM, parental HRQoL and family functioning were lower in all sub-scores in this more severe cohort [1, 7]. Scores were similar to one other study in a severe cohort [21]. HRQoL and family functioning scores were not higher in parents of older children, despite improvements in children’s physical health, including the decreased respiratory morbidity and more positive parental assessments of the child’s overall health, that we have previously reported [11]. A diagnosis of autism, a feeding tube, and having wheezed in the past year were most strongly correlated with HRQoL total scores, though many other respiratory and developmental outcomes also had important impacts on the family. Three elements of our findings warrant further discussion.

First, parents of older children did not report improved parental HRQoL and family functioning compared to parents of younger children, as would be consistent with studies in less severe BPD cohorts [1, 7–9]. This finding suggests that adaptation and improved respiratory symptoms seem insufficient to better family life. A plausible explanation is that although physical health of the children improves, the neurodevelopmental sequalae weigh more heavily on parents as the child ages. Nearly half of our participants were told by a doctor that their child had developmental delay and almost all of the children required an IEP for school [11]. Autism and developmental delay were also some of the most strongly associated outcomes with both parental QoL and child QoL in our cohort [11]. Problems with development may also lead parents to worry about future academic success and their children’s’ potential to gain independence, further impacting QoL. The shifts in the specific questionnaire items that were most problematic suggest a shift from difficulty with household tasks to concerns about how their child will engage with society and how others will react to their child’s disabilities. The severity of disease in this cohort likely explains the discrepancy between our reported stagnation of family outcomes, and other studies reported improvement. The impact on parents of school-age children with such severe BPD sequelae has not previously been reported.

Second, we found that many medical factors negatively impacted parental HRQoL and family functioning, presumably by increasing the burden of caring for children with severe BPD. Our findings that the severity of respiratory disease correlates with parental HRQoL and family functioning are consistent with prior work done in more mildly affected populations [1, 7]. However, our data suggest that respiratory morbidities do not convey the whole story, as autism and other developmental sequelae which are far more common in patients with severe BPD also have a major impact on families. While tracheostomies have often been used as an indicator of severe disease, they are no more correlated with parental HRQoL than the ability to walk or developmental delay. Surprisingly, however, tracheostomies were correlated more strongly with parents’ report of their own HRQoL than parent-reported child HRQoL. This is consistent with some prior work and further supports the hypothesis that while the parent’s perceived impact of the tracheostomy on the child was minimal, parents themselves were overwhelmed by the pressure of caregiving, were experiencing adverse effects on their sleep, and were worried about their child’s health after tracheostomy [22]. We were surprised to find that CP, poor weight gain, vision and hearing impairment were not associated with PedsQL FIM. Perhaps conditions that are stagnant rather than progressive or episodic better allow for parental adaptation. Overall, our findings suggest researchers should measure a wide variety of outcomes—rather than rely on respiratory morbidity— to capture the effect of severe BPD on families. Additionally, these results demonstrate the importance of clinicians understanding and addressing the many ways that a child’s clinical trajectory impacts parents over time.

Third, many of the issues cited as most problematic to family HRQoL are modifiable with additional support, particularly financial support. These issues include feeling tired, feeling like people don’t understand their situation, and lacking time for chores and social activities. Providing additional resources for families, such as access to respite care, home nursing, medical daycare, support groups and counseling has the potential to substantially improve the experiences of parents of children with BPD. Past studies have found that parental income in families of chronically ill children predicts positive quality of life and that peer support groups are helpful in reducing distress [23, 24]. Additionally, home nursing is highly valued by these families [25, 26]. Increasing access to resources like financial support and nursing for families of children with severe BPD would likely improve familial HRQoL and functioning. Several studies have demonstrated that increased family functioning leads to improved adherence to medical regimens and control of symptoms in children with chronic conditions [27–29].

Our study design has strengths and limitations. First, our cohort consists of children referred to a specialized program at a single center. However, this is also a strength because given the severity of BPD required for referral to the NeoCLD program at CHOP, our patient cohort provided an opportunity to study parental HRQoL for a common condition in its most severe form. This data is applicable to other centers that care for patients with severe disease. Second, our data collection overlapped with the beginning of the Coronavirus-19 pandemic, which could have led to further challenges for the families. However, our data largely represents outcomes prior to this period. Another limitation is that our data on post-discharge diagnoses are gathered through parental report rather than medical records. Still, it would be unlikely for parents to misremember important diagnoses, especially to overestimate. Another limitation is that our study was cross sectional rather than longitudinal, so age-related differences may in part reflect changes in standard care over time. Additionally, given that we interviewed each family at one moment in their lives, the circumstances outside of their child’s health status at that time may have influenced their responses. Finally, our response rate was relatively high for a follow up study [7, 30], and the baseline similarities between responders and non-responders are reassuring against sampling bias (Supplementary Table 1).

## Conclusion

The present study captures the impact of severe BPD on parental HRQoL and family functioning. While the physical health of these children improves with age, and the nature of families’ concerns shift, parental HRQoL and family functioning are not improved in families with older children. This highlights the importance of evaluating and addressing not just respiratory morbidity in this population, but also the neurodevelopmental outcomes and healthcare utilization needs that continue well into childhood. Despite past evidence that parental HRQoL of children with BPD improves over time, our results demonstrate that families with particularly severe disease and significant neurodevelopmental sequalae experience continued impact throughout childhood. Additionally, these results suggest that families have different concerns and reasons for decreased HRQoL depending on the age of their children. Many of the parents’ specific concerns and struggles were modifiable. The data collected in this study can be used by physicians to identify the specific sequalae of BPD that have the largest impact on families and target those families for additional support. That concerns vary by age suggests that the most effective interventions may also vary by age and longitudinal follow up by expert providers in how severe BPD evolves over time is of the utmost importance. An enduring question is which interventions will most effectively target parental quality of life in these patients and what indicators can be used to predict the most effective interventions.

## Supplementary information

Supplemental Tables and Figures

## Data Availability

All data is available upon request, which should be submitted to the corresponding author.
