# Supplementary material for: Health-related quality of life among families of children with severe bronchopulmonary dysplasia
Source: J Perinatol. 2025 Nov 4;46(2):200–5. doi: 10.1038/s41372-025-02468-x (PMC12909121; doi:10.1038/s41372-025-02468-x)
Supplement: Supplementary file 1 — Supplemental Tables and Figures [file 41372_2025_2468_MOESM1_ESM.docx]

**Supplemental Tables and Figures**

**Supplemental Figure 1. Enrollment flow chart.**


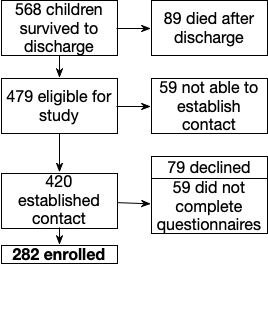


**Supplemental Table 1. Characteristics of respondents versus non-respondents.** Comparison between respondents and non-respondents, families who were eligible and reachable but did not complete the study. P-values reflect comparisons using t-tests.

| **Characteristic** | **Respondents Mean (SD) N=282** | **Non-Respondents Mean (SD) N=138*** | **p-value** |
| --- | --- | --- | --- |
| Birth weight (g) | 839.2 (458.6) | 830.7 (477.1) | 0.42 |
| Gestational age (weeks) | 26.0 (2.7) | 26.1 (2.9) | 0.42 |
| Gestational age at discharge (weeks) | 61.6 (14.3) | 60.3 (12.9) | 0.17 |
| Age at attempted contact (years) | 5.7 (2.9) | 6.0 (3.0) | 0.17 |

**Supplemental Table 2. Correlations between outcomes vs. PedsQL FIM total score at follow up.** Bold text indicates significance

| **Characteristic** | **Change in PedsQL FIM (Unadjusted)** | **P-Value** |
| --- | --- | --- |
| **Autism** | **-11.34** | **p<0.001** |
| **Feeding tube** | **-11.10** | **p<0.001** |
| **Wheezing (Past 12 months)** | **-10.36** | **p<0.001** |
| **Developmental delay** | **-9.61** | **0.001** |
| **No respiratory support (room air)** | **6.45** | **0.025** |
| **Ability to walk alone** | **8.07** | **0.007** |
| **Parent reported health** | **-7.36** | **p<0.001** |
| **Tracheostomy** | **-7.94** | **0.001** |
| **Respiratory medications (Per additional)** | **-4.60** | **p<0.001** |
| **Medications (Per additional)** | **-3.50** | **p<0.001** |
| **Therapies (Per additional)** | **-3.91** | **p<0.001** |
| **Rehospitalizations (Per additional)** | **-2.01** | **p<0.001** |
| Cerebral Palsy | -5.92 | 0.11 |
| Poor weight gain | -3.67 | 0.12 |
| Vision loss | -1.36 | 0.58 |
| Hearing Loss | 0.96 | 0.87 |
